# Counting insects

**DOI:** 10.1098/rstb.2016.0513

**Published:** 2018-01-01

**Authors:** Peter Skorupski, HaDi MaBouDi, Hiruni Samadi Galpayage Dona, Lars Chittka

**Affiliations:** 1 Institute of Medical and Biomedical Education, St George's, University of London, Cranmere Terrace, London SW170RE, UK; 2 School of Biological and Chemical Sciences, Queen Mary University of London, Mile End Road, London E1 4NS, UK; 3 Wissenschaftskolleg, Institute for Advanced Study, Wallotstrasse 19, D-14193 Berlin, Germany

**Keywords:** bee, brain size, counting mechanisms, neuronal number, numerical cognition, working memory

## Abstract

When counting-like abilities were first described in the honeybee in the mid-1990s, many scholars were sceptical, but such capacities have since been confirmed in a number of paradigms and also in other insect species. Counter to the intuitive notion that counting is a cognitively advanced ability, neural network analyses indicate that it can be mediated by very small neural circuits, and we should therefore perhaps not be surprised that insects and other small-brained animals such as some small fish exhibit such abilities. One outstanding question is how bees actually acquire numerical information. For perception of small numerosities, working-memory capacity may limit the number of items that can be enumerated, but within these limits, numerosity can be evaluated accurately and (at least in primates) in parallel. However, presentation of visual stimuli in parallel does not automatically ensure parallel processing. Recent work on the question of whether bees can see ‘at a glance’ indicates that bees must acquire spatial detail by sequential scanning rather than parallel processing. We explore how this might be tested for a numerosity task in bees and other animals.

This article is part of a discussion meeting issue ‘The origins of numerical abilities’.

## 1. Introduction

‘Two tigers were seen going into the cave. Only one came out. Is the cave safe?’ This stark example [1] illustrates the survival value of a non-verbal, non-symbolic sense of number. Predator vigilance, foraging and navigation are obvious contexts in which ability to assess quantity would seem adaptive. The more complex the interaction with the environment, the more likely it is that an organism will benefit from estimating and keeping track of quantitative variables, including time and magnitude (countable and non-countable). The basic operations of cognition track both objects and events in order to make appropriate decisions. Arguably, however, there has been a tradition to view cognitive processes as distinct from ‘simple’ associative learning. Undeniably, humans engage in higher cognitive processes during mathematical reasoning or when thinking about temporal relations and causes. Yet as long ago as 1946, arguing from the results of a series of influential experiments, Michotte proposed that causality is a basic attribute of visual perception [2]. More recently, the same has been argued for perception of numerosity [3]. Nevertheless, there remains a tendency to fetishize numerical cognition, because of its association with the most advanced human intellectual achievements. Consequently, demonstrating any form of numerical competence in non-human animals requires tortuous controls, to rule out discrimination on the basis of some continuous magnitude rather than numerosity *per se*. These controls are indeed required, but carry the implicit assumption that quantity discrimination is inherently more complex for countable rather than non-countable quantities, perhaps reflecting a higher cortical function. Nevertheless, numerosity discrimination has been demonstrated in vertebrates that lack the mammalian neocortex (see Agrillo & Bisazza [4]). In fact, it seems highly unlikely that the architectural plan of the vertebrate brain is necessary for basic numerical cognition; cuttlefish, for example, have recently been claimed to discriminate prey items on the basis of numerosity [5]

Contrary to the notion that numerical cognition is a complex, higher cortical function, theoretical studies indicate that numerical discrimination requires no more than a classifier and a threshold mechanism [6], which can be implemented by known neural circuits ([7]; see also Rose [8]). The extent to which such mechanisms can explain numerical cognition remains to be determined, but the point is that we should not necessarily be surprised that cognitive animals can keep track of entities in their environment, including, up to a point, number of entities. The question of how they do this is of central neurobiological interest, which involves more than demonstrating proto-human counting abilities in animals. Here, we review the literature on counting-like abilities in insects. We argue that there might be relatively little mileage in discovering more animal species with numerosity capacities, since the ability in itself might be relatively trivial. A promising avenue of future research might be to explore *how* animals such as insects solve numerosity tasks, which requires a detailed inspection of their choice behaviour rather than just tallying correct versus incorrect choices in discrimination tests. Such an exploration might reveal that insects (and perhaps other animals) count by fundamentally different strategies, underpinned by different mechanisms, compared to humans. Specifically, the need to acquire visual-spatial information by sequential scanning, rather than parallel processing of entire visual scenes, might require insects to inspect items one after another, and limit their ability to subitize (seeing numbers at a glance).

## 2. Numerical cognition in invertebrates

Compared to comparative studies in vertebrates, rather less is known about numerical cognition in invertebrates. However, it is clear that both countable and non-countable quantitative information may be used in guiding behaviour. Ants, for example, measure distance by integrating step count [9,10] but can also learn to use size of visual stimuli as direction cues [11]. Bees can perform visual discrimination on the basis of both size [12] and numerical quantity [13].

An early exploration of numerosity in bees was performed by Leppik [14]. This is a useful case study in the adaptive utility of a number sense, as well as the pitfalls that need to be avoided when studying whether subjects respond to number rather than other cues that would allow the same outcome. Leppik noted that radially symmetric flowers often have relatively low numbers of petals (e.g. 3,5,6 or 7) and suggested that bees might remember the species-specific number of petals to distinguish rewarding from unrewarding species. To support his idea, he removed defined numbers of petals from some flower species and monitored bee visitation rates before and after the manipulation. He found that bee visits were substantially reduced when petal numbers were lowered, and concluded that bees must have been sensitive to petal number. This is possible, but without control tests, it is equally plausible that bees might instead have responded to reduced contour length, area subtended, or they might have been deterred by odour cues emanating from damaged flowers.

**Figure 1.**
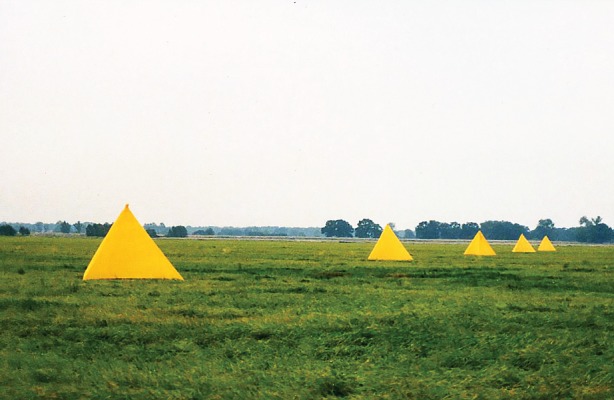
Landmark counting by honeybees in an open field. Bees were originally trained to fly from a hive (out of view to the left) to a feeder located at a distance of 262.5 m, between the third and fourth of a series of yellow tetrahedral tents, spaced 75 m apart. In subsequent tests, spacing between the tents was systematically varied and two feeders were offered; one at or close to the distance from the hive learned during training, and a second spaced between the second and third tents, and consequently, at an altered flight distance from the hive [15]. The question was, would the bees be more likely to find the feeder at the trained distance, or would they find it by the number of landmarks passed during training flights? See text for details.

Chittka & Geiger [15] provided the first evidence that numerical cues may be used in honeybee navigation. Bees were trained to forage from a feeder in an open field located at a fixed distance from the hive (262.5 m). A series of yellow, tetrahedral tents of 3.5 m height was set up, to act as landmarks along the flight path (figure 1). The feeder was located midway between the third and the fourth landmarks. Following training, bees were tested in a control experiment, where a second feeder was placed closer to the hive, between the second and third tents. In this situation all but one of the bees flew the original distance to the trained feeder. Next, the relationship between flight distance and number of landmarks passed was systematically probed by varying the number of landmarks and the distance between them. For example, in one test the spacing between tents was decreased so that the trained feeder was now located between the fourth and the fifth tents. A second feeder was located between the third and the fourth tents, at a shorter distance from the hive. Would the bees choose to fly the original distance, past four landmarks instead of three, or would they choose the feeder located at a shorter distance but past the previously experienced number of landmarks? Most of the bees (76%) landed at the feeder located closest to the trained feeder, but a quarter landed at the test feeder between the third and fourth tents. In further tests where the number of landmarks were increased or decreased, the bees' group behaviour suggested a compromise between an estimate of the learned distance and landmark cues in test feeder choice. However, in all cases, a significant minority (8–26%) of bees based their landing decisions on the number of landmarks (i.e. choosing a feeder located between the third and fourth tents regardless of distance). Since no transfer to other types of countable objects was explored, Chittka & Geiger argued that a ‘proto-counting’ strategy was the likeliest explanation for the behaviour of this group of bees.

**Figure 2.**
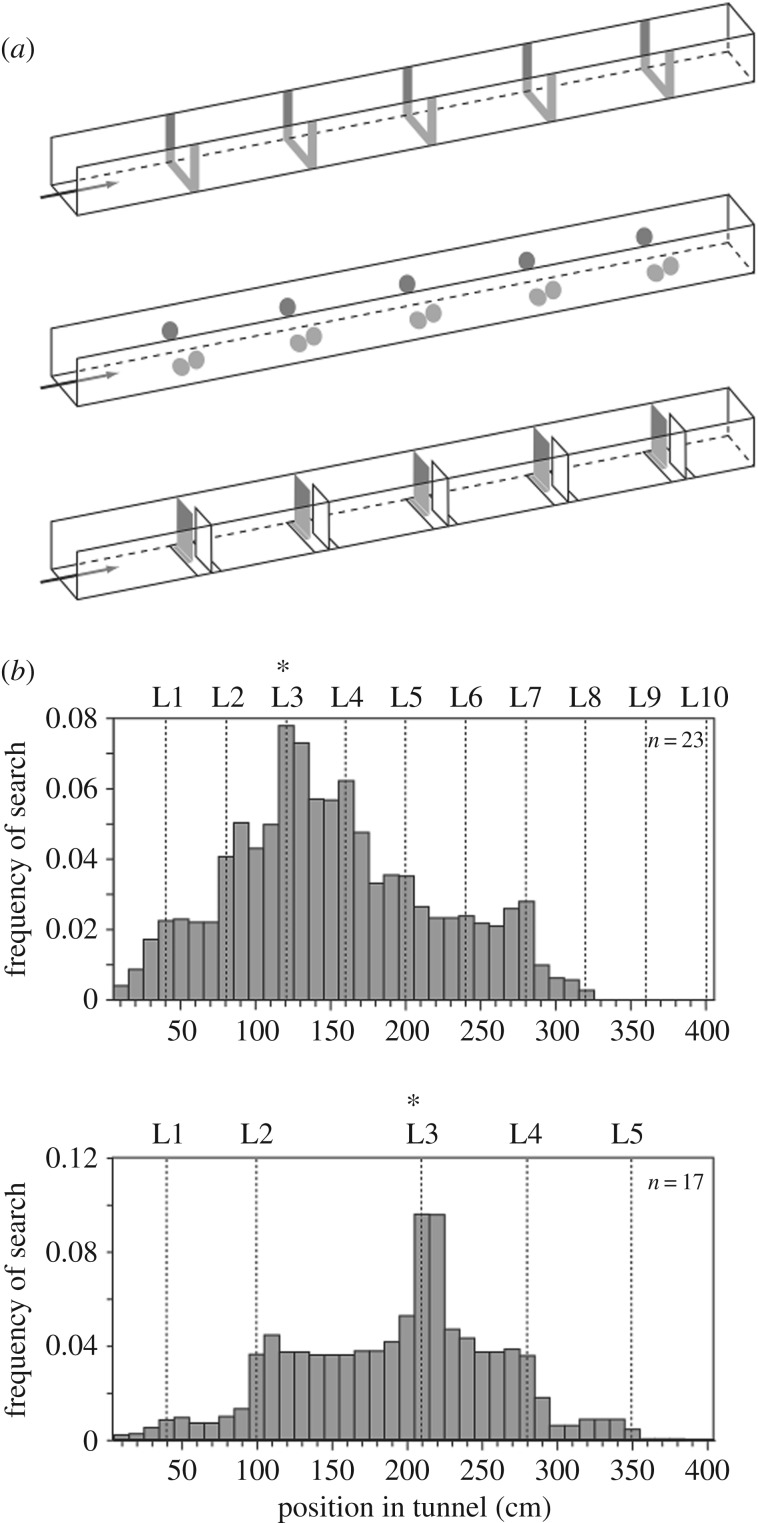
Landmark counting in a laboratory flight tunnel. (*a*) Individual bees were trained to receive a reward after they had flown past a specified number of landmarks. During training, the landmarks were strips of evenly spaced yellow paper (upper). Spacing interval was randomly varied every 5 min, to ensure the bees could not learn the reward location by measuring flight distance. Different experimental groups were tested on the same landmarks as in training; in tunnels where the stripes were replaced by yellow disks, presenting a smaller cumulative yellow surface; or in tunnels where landmarks were arranged as baffles, so that only one could be seen at a time. (*b*) Results of an experiment where bees were trained on landmark 3, then tested with landmarks spaced regularly every 40 cm (upper panel) or irregularly spaced (lower panel). Modified from Dacke & Srinivasan [21], with permission.

At the time of the discovery in the mid-1990s, this result seemed rather startling. Although the associative learning abilities of bees were not in doubt, counting was viewed as a ‘higher’ cognitive function, beyond simple association ([16], but see [17,18]). However, the Chittka & Geiger result has since been replicated by multiple teams, including a field study, where harmonic radar was used to track the choices of individual bees [19]; again, the majority of bees based decisions on distance flown, but the search behaviour of a clear minority was centred on the landmark number that had previously cued feeder location. This suggests that the honeybee's odometer (distance estimator) dominates navigation learning, which is perhaps not surprising, since it is distance that is communicated to nest-mate foragers via the waggle dance [20]. However, analysis of the behaviour of individual bees indicates that sequential, countable cues are also learned. This point has been confirmed and extended in a controlled laboratory setting [21], which allowed cue manipulation to demonstrate unambiguously that the bees were learning numerosity *per se*. The investigators were also able to determine the upper limit for bees' numerical representation in this task, which appears to be a maximum of four landmarks. Importantly, it was also shown that the bees could abstract numerosity from the particular perceptual details of the stimuli, as if learning a rule ‘search after three’ irrespective of the particular cue used (figure 2). This ability to abstract numerosity in transfer tests is regarded as a key component of numerical cognition [22].

**Figure 3.**
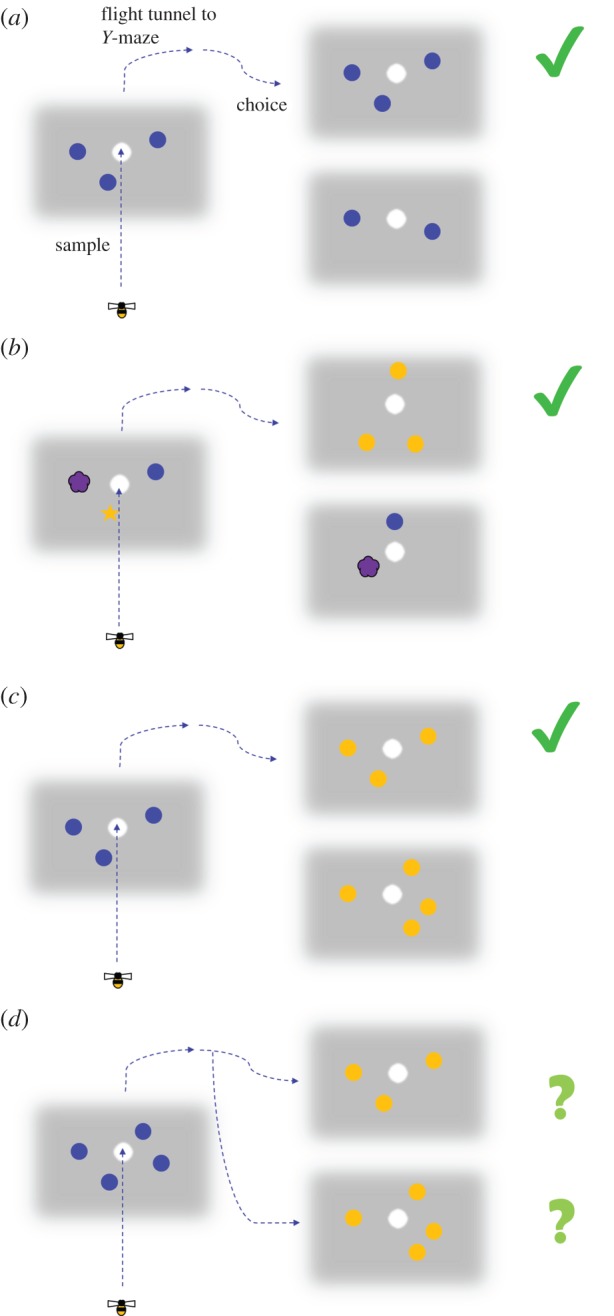
Summary of bees' choice behaviour in the experiments of Gross *et al.* [23]. Following training on either two or three stimuli, bees were tested in discrimination or transfer tests with a sample of numerosity either two or (illustrated here) three (in the actual experiments the correct arm of the *Y*-maze was randomized). (*a*) Exact pattern match. (*b*) Pattern matching by numerosity only. Size, configuration, colour were varied in extensive series of transfer tests to rule out non-numerical cues. (*c*) Bees were able to match to sample when distractor contained novel numerosity (four), but performance was not significantly above chance when the sample contained the novel numerosity (*d*). Bees were also unable to discriminate between stimuli containing four and six items (not shown).

The ability of bees to generalize visual stimuli purely on the basis of number was probed further in a carefully controlled study by Gross *et al.* [23]. Bees were trained on a delayed-match-to-sample task where the matching required learning the number of elements in the visual stimuli. Initially bees were trained on a sample of either two or three dots and required to choose the matching sample from the appropriate arm of a *y*-maze (figure 3). This task was readily learned. Extensive control experiments varied the orientation, colour and shape of the individual elements of the stimuli to minimize the possibility that the bees could solve the task on the basis of anything other than abstracted numerical quantity. Importantly, the bees were able to generalize the match-to-sample rule to novel stimulus items, but the limit for this was between three and four.

In an interesting exploration of counting in an ecological context, the behaviour of bumblebees foraging from flowers with five nectaries was analysed [24]. Optimal behaviour here would be to avoid revisiting depleted nectaries, which implies keeping a tally of number visited, and not visiting more than five. In the field, the probability of departing from a flower increased sharply with number of nectaries probed up to the number of five, and this number of nectary probes was by far the most common. A sixth nectary probe (i.e. a revisit) was very rare. The authors were able to exclude alternative explanations, for example that bumblebees used scent marks left by their tarsal glands to avoid nectaries already visited. Solitary bees (*Eucera* sp.) also mastered this task, but with less precision and in a manner such that the authors could not rule out alternative explanations, such as using scent marks or simply abandoning flowers when bees encountered empty nectaries [25].

In a controlled laboratory experiment, it proved rather difficult to train bumblebees to artificial flowers that would reward only two probes; over 1000 trials were required before the bees learned to depart after two to three probes [24]. The authors note that the nectaries are only visible to the bees one at a time, and largely indistinguishable based on visual features. Consequently, a form of motor sequence learning may have been required to keep tally of number of probes.

Recent evidence suggests that orb-web spiders maintain a tally of prey items in ‘larders’, which they accumulate on their webs [26]. Removal of the prey larder elicits searching behaviour, the duration of which is proportional to larder size, suggesting spiders remember the size of the larder they have accumulated. Rodríguez *et al*. [26] attempted to assess the relative roles of prey count as against total quantity. Search time increased both with prey mass and number (up to four prey items), but the rise was steeper for increasing prey count compared to equivalent increases in mass with individual prey items, suggesting the spiders kept track of prey numerosity.

## 3. Systems for number representation

Human numerosity discrimination may involve counting, estimation or subitizing [27]. Counting, in the strictest sense of the word, requires a symbolic number system (numerals), developed in some human cultures [28]. In animals, counting-like abilities are said to exist where a response to the number of stimuli in a set can be abstracted to qualitatively different sets of stimuli [29,30]. Subitizing is the ability to perceive the number of items in a small set, which is accurate up to about four items (and in humans is accomplished ‘at a glance’). Estimation is the ability to judge approximately the numerosity of larger sets without counting. In comparative studies too a distinction is commonly made between numerosities consisting of four items or fewer (the subitizing range), and larger numbers [22,31]. The large number system (estimation) is analogue and approximate and the error around the test number scales with its magnitude, according to Weber's law. This entails that the accuracy with which two numerosities can be discriminated is limited by the ratio of their size difference rather than the absolute size difference. The small number system, by contrast, is exact, ratio independent and has an abrupt limit of three or four items [31]. It is possible that these two number systems are a basic feature of vertebrate cognitive architecture since it has been demonstrated in both guppies and college students [32]. It may be significant that an upper limit of around four items has also emerged from the recent studies on bees reviewed above [21,23]. The existence of a ratio-dependent system for approximate comparison of larger numerosities has not been found in invertebrates (at least for visually based decisions). However, male mealworms were shown to keep track of number of females in olfactory bouquets from up to four females; there was a ratio-dependence greater than 1 : 2 in this range; males could discriminate one from three or four, but not one from two, or two from four [33]. It should also be noted that non-countable quantity estimation *has* been shown to be subject to Weber's law in invertebrates. The visual odometer of the honeybee, for example, estimates distance by integration of retinal flow of visual texture [34], and in experiments where bees are trained to fly a set distance, the error is proportional to the trained distance [35].

The symbolic number system made possible by human language eases the constraint imposed by Weber's law. Given number symbols (words), arbitrarily large numbers can be discriminated with equal accuracy. For example, discriminating 109 from 110 would be impractical (though not formally impossible) for an analogue approximate number system that was not also mapped to a symbolic number system. Nevertheless, human reaction times in discriminating numerals such as 109 from 110 would be expected to be longer than discriminating 110 from 190; such a ratio-dependent effect is a signature of an analogue system [36]. However, with an analogue magnitude system mapped to a symbolic number system, arbitrarily large numbers can be discriminated with the same accuracy (albeit with a speed–accuracy trade-off) as small ones, and indeed without the need for an increased working-memory capacity. All that is needed is to keep track of the last number counted, and a spatial counting strategy to avoid counting items twice (e.g. left to right plus top to bottom in a vertical 2D display). Without word labels, counting to higher numbers is inherently much more challenging. It has in fact been suggested that the development of uniquely human cognition involved an evolutionary trade-off between working memory and symbolic representation capacities [37].

## 4. What accounts for the upper limit of around four countable items in many species?

If it is indeed the case that a small number system is based on object individuation—the representation of distinct objects—then arguably it may be a fundamental attribute of cognition and perception [31] and subject to the capacity limit of working memory, classically assumed to be in the range 4–7 [38], but more recently argued to be centred around four items [39]. What accounts for this limit?

One possibility is that the limit is inherent to the dynamics of neural circuitry [40]. An influential, if unproven, hypothesis is that objects are represented by neural assemblies, which bind local sensory features into coherent percepts [41–43]. For example, edges at disparate spatial locations could be part of one large or two smaller objects. The same applies to other visual attributes: a yellow star, blue triangle and green circle are three items, which have to be individuated incorporating differences in colour, shape, size, etc. Spike synchrony has been proposed as a mechanism to do this. To bind ‘green’ with ‘triangle’ requires the spiking signals for green (but not those for blue) to be synchronized with those for the triangle. A large body of work suggests that this synchrony is achieved by means of neuronal oscillations with a frequency of about 40 Hz; neurons belonging to the same assembly would oscillate in phase and thus have a strong tendency for synchronous spiking. In our simplistic example, neurons signalling blue would not fire in phase with those signalling triangle and would thus be considered to belong to a different assembly. Note that the individual sensory signals are independent of these phase relationships. The spike count over an integration window can be similar, with or without synchronization to other neurons. Synchrony mediated by oscillation functions as a carrier signal to assign neurons to assemblies, rather than as a code for any particular perceptual attribute. Numerosity, according to this scheme, is not coded by any particular feature that defines a neural assembly (such as phase with respect to oscillation cycle); rather, numerosity is inherent in the number of assemblies that are active.

A clear implication of this is that there will be a trade-off between the number of neuronal assemblies to be maintained simultaneously, and the stability of each representation. This will be based on the fundamental temporal dynamics of the neuronal membrane. A larger number of simultaneously active assemblies means smaller phase differences between each assembly. The accuracy with which neuronal spiking can be timed to phase will therefore impose a maximum on the number of assemblies that can simultaneously be maintained before the assignment of a particular spike to a particular assembly becomes ambiguous (and therefore, it may no longer be possible to distinguish blue triangle and green circle form green triangle and blue circle). Simulation studies suggest this limit is in the range of 4–7 [40]. In principle, any coding scheme based on dynamical neural assemblies will be constrained by the temporal resolution of individual neurons.

The precise role of synchronous oscillations in defining functional neural assemblies is a matter of continuing debate, although a functional role is reasonably well established in the olfactory system of both mammals and insects. Oscillations in the 10–30 Hz range have been recorded from locusts [44], honeybees [45] and *Drosophila* [46] and have been shown to be necessary for fine odour discrimination in locusts and honeybees.

The known temporal dynamics of insect and mammalian brains operate over similar time scales, in contrast to the known dissimilarities in brain architecture, and orders-of-magnitude differences in neuronal number. This implies that the difference between the large brains of primates and the small brains of bees might be in representational richness, not in the number of separate representations that can be simultaneously maintained. Brain size will have an impact on the size of neuronal assemblies (more neurons available for each assembly) and therefore the amount of information that can be processed in parallel, but not on the number of neuronal assemblies that can simultaneously be maintained (which would be constrained by similar temporal dynamics in large and small brains). Bigger brains allow more parallel processing [47,48].

## 5. Numerical cognition in small brains

Is it surprising that numerical cognition in animals is independent of the crowning glory of mammalian neocortex? Probably not [49,50]. An influential model [6] suggested a rather simple mechanism for extraction of numerosity from magnitude. Indeed, this model consisted of three modules containing a total of 530 independently firing units (neural clusters in their case, but for functional purposes they might be regarded as individual neurons) and with this limited tool kit, the network could extract approximate numerosity from parallel visual displays (up to five items in this case, although in principle this is not limited, but depends on the size of the input array). The variance in the numerosity estimate in this model increased in proportion to the numerosity itself (Weber's law); in keeping with this the model could reliably discriminate two from three, but was only slightly above chance for three from four [6], a performance similar to human infants [51]. Nevertheless, this suggests that even if an insect evolved a dedicated small number discrimination module *de novo*, without capitalizing on abilities emerging from existing circuits [52], the added 500 neurons would hardly be detectable in terms of gross neuroanatomy even in a brain as small as *Drosophila*'s. More recently, a deep learning algorithm, containing just two hidden layers with 35 neurons, was able to model successfully key results from human and non-human animal studies [53]. This mirrors other studies in computational neuroscience which show that the single task that requires a big brain, in terms of the computational capacities required, remains to be discovered [49]. Clearly, large brains are not a prerequisite for numerical cognition.

A fundamental misunderstanding in cognitive neuroscience may be that in order to discriminate by a certain visual attribute, one needs to have a specialized neuron type for it (the neuron doctrine). However, so long as a certain visual feature (be it number, area, edge orientation, symmetry, texture, etc.) reliably activates an identifiable ensemble code of multiple neurons, that feature can be encoded—and thus, learned about. It may indeed be the case that neurons can be shown to respond to approximate numerosity [54], among other things, but this does not mean that numerical cognition, in the first instance depends on specification of numerosity-detecting neurons. Consider, for example, that the optic lobes of insects (lamina, medulla, lobula (and lobula plate, in some insects)) contain perhaps 200 neuronal classes, and together comprise approximately half of the brain [55,56]. Although many optic lobe neurons have historically been described as e.g. ‘colour coding’ [57,58], ‘orientation detecting’ [59], ‘motion coding’, most have in fact extremely complex response properties, responding to a wide variety of stimuli, depending on eye region, spectral content and behavioural context, and may at best be described as responding *predominantly* to a certain stimulus attribute. Recently, it was shown that a neural network with as few as eight of the simplest feature detector neurons was able to discriminate a large variety of seemingly complex visual patterns that had previously been used in honeybee learning experiments [60]. In reality a subset of some 200 000 Kenyon cells of the mushroom bodies will be sampling the output of perhaps 200 classes of optic lobe neuron [61]. So long as any stimulus property (e.g. number) is represented by a recognizable ensemble code at the interface between these projection neurons and the mushroom body intrinsic Kenyon cells, that stimulus property is codable and memorizable. In this sense, number might simply be an emergent property of an ensemble of neurons with even a modest diversity of response properties.

## 6. Subitizing—counting at a glance?

Subitizing is the ability to recognize the number of items in a visual scene without the need for sequential counting. This ability is limited to around four items. Unlike the approximate (analogue magnitude) number system for assessing large numerosities, where discrimination accuracy depends on the ratio of two-numerical magnitudes [22], subitizing is thought to be exact, and is thus not expected to show a ratio-dependence. In addition, it involves rapid, parallel assessment of object items. Field observations and spontaneous choice experiments have often suggested an upper limit of around four items in a variety of species, while with laboratory training larger numbers can be discriminated, suggesting an analogue magnitude system. This in itself, of course, does not prove that distinct number systems are used in the two types of tasks [62]). For example, although untrained cuttlefish can discriminate one prey item from two and, in steps of one, up to four from five, the decision time increases monotonically as the ratio difference decreases, which is not what would be expected if a subitizing mechanism was responsible for the discrimination of small numerosities [5]. Such increases in response time with number of items to be processed indicate serial, rather than parallel, evaluation of the visual scene [63,64].

In insects, at least, limits on the parallel processing of the visual scene may be expected on the basis of fundamental constraints imposed by compound eye design. The eye of a bumblebee, for example, consists of 3000–4000 ommatidia [65] and visual acuity is limited to around one degree of visual angle [66], which seems to compare very poorly with the 2 million cones and 0.5 arc min resolution of the primate visual system. However, in terms of temporal resolving power, the primate cone is outperformed by the photoreceptors of many species of fast-flying insects. The fastest known physiological response of any ocular photoreceptor was recorded from the blowfly: at 34°C the impulse response begins at around 3 ms, peaks at 6 and is complete by 10 ms [67]. This is reflected in flicker fusion frequencies, which reach a maximum of 70–80 Hz under optimal conditions with human observers, but are around 200 Hz in bees [68] and possibly even higher in some flies.

**Figure 4.**
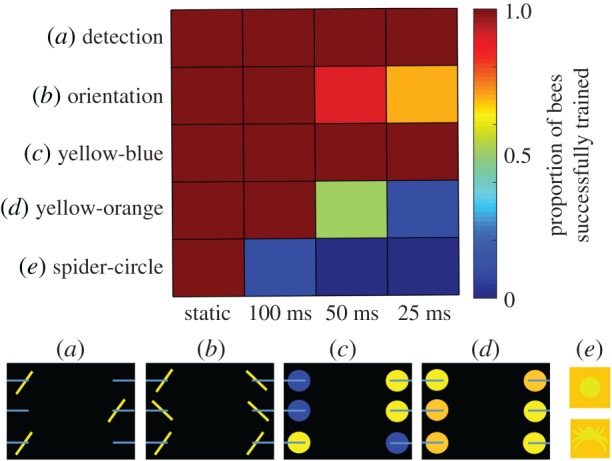
Bees cannot perceive complex visual stimuli ‘at a glance’. Bees were trained in a flight arena with six feeding platforms (blue horizontal lines in the panels on the bottom) positioned in front of a 120 Hz (8.33 ms refresh rate) gaming monitor. Separate groups of bees were trained on five tasks, ranging from simple detection to complex pattern discrimination, in four temporal presentation conditions, ranging from continuous presentation (static) to timed presentation (repetition rate randomized between 500 and 1000 ms) for 100, 50 or 25 ms. (*a*) Detection of oriented bar; (*b*) discrimination of 45° from −45° bars; (*c*) coarse colour discrimination yellow-blue; (*d*) fine colour discrimination yellow-orange; (*e*) discrimination of spider shape from circle (only two of six stimuli shown for simplicity). All of the bees were successful in acquiring the simple detection task, regardless of presentation duration. For fine colour discrimination, stimulus durations of at least 50–100 ms were required (*d*), while only a single bee learned the shape discrimination at 100 ms, even though all bees learned the task under continuous presentation (*e*). Modified from Nityananda *et al.* [47], with permission.

Bumblebee photoreceptor processing speed also easily outperforms that of primates [69]. But high-performance photoreceptors do not come cheap: the short membrane time constants required for temporal precision are attained by substantial increases in membrane conductance. This incurs a substantial metabolic cost, largely due to the energy expenditure required to maintain concentration gradients in the face of large conductance increases; crepuscular or less rapidly moving species forego this expenditure [70–72]. What justifies this expenditure in the case of worker bees? A major effect of increased temporal resolution is to reduce motion blur. If a serial strategy, possibly depending on active vision, is used by bees, then fast photoreceptors would increase the information extracted from fast, brief scanning movements. It also implies that bees would be unable to extract complex visual information from a static sensory snapshot. In support of this hypothesis, it was recently shown that bees fail all but the simplest visual discrimination tasks when stimulus presentation duration is limited ([47]; figure 4). This is similar to the situation in tethered bees, which can learn visual discriminations of simple, large colour or stimuli [73,74], but have not yet been shown capable of complex visual discrimination, which is just what we would expect if active visual scanning is required in the latter case

The subitizing mechanism is often taken to be ‘seeing at a glance’ and indeed, in experiments on humans and non-human primates visual presentations are often very brief to ensure this. However, parallel as opposed to sequential presentation of visual stimuli does not necessarily lead to parallel as opposed to sequential processing by the nervous system. In the experiments on number-based visual generalization in honeybees [23] timing data for the bees' choices are not provided, although the authors do note that in transfer tests the bees appeared to spend additional time scanning the stimuli where the target and distractor numerical quantities were presented via elements with novel perceptual qualities. In fact this scanning behaviour itself, we suggest, may hold the key to understanding how bees come to make the numerical discriminations they do. One possibility is that coding via motor sequences [75] may be involved. Additionally, bees could exploit the high temporal resolution of their vision in order to extract additional spatial information in an active vision strategy, if the motor commands of scanning movements could be correlated with precisely timed visual information. The latter proposal would be more difficult, but not impossible, to test; either way we suggest that focusing on the temporal dynamics of bees' visuomotor search behaviour will help reveal the underlying basis of their numerical discrimination.

**Figure 5.**
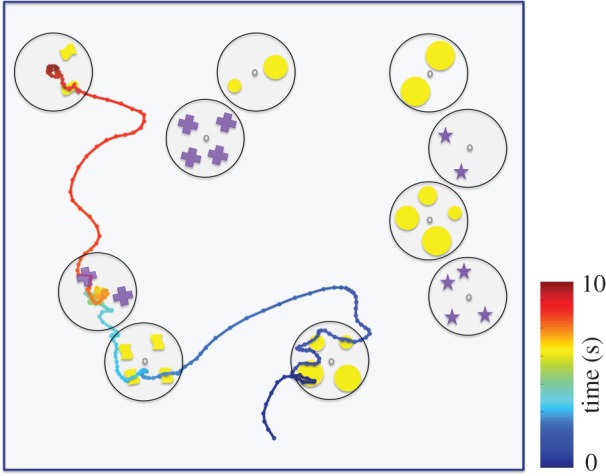
Flight path of a bee trained, with differential conditioning, to select stimuli with two items and avoid those with four. The first 10s of the bee's scanning behaviour are shown; the path is colour-coded to show the progression from early (violet) to late (red). The bee sequentially examines two patterns containing four items, but rejects each of them after scanning three items in each. She then chooses a pattern containing the correct number of two purple crosses (even though she has not been rewarded on any other dots than yellow ones before) and finally selects another pattern with the correct number of two (yellow) dots. Dots are separated by time intervals of 33 ms. See also electronic supplementary material, video S1.

In one preliminary experiment, using differential conditioning, a bumblebee was rewarded on various patterns containing two elements, and trained to avoid patterns that contained four (figure 5; electronic supplementary material, video S1). Perhaps unsurprisingly, the bee learnt the task independently of the colour or shape of the elements, or the area subtended by them. The flight path of the bee holds interesting clues to the decision-making and counting process. The bee inspected and scanned both ‘twos’ and ‘fours’, indicating that it could not make a decision from a distance. The flight path shows the bee inspecting items within a pattern one by one, similar to the kind of ‘motor tagging’ observed in some primates [76]. The bee avoids scanning the same stimulus element multiple times, indicating working memory control of the scanning behaviour. However, after scanning, the bee not only landed more frequently on ‘two’ than ‘four’, but also rejected more ‘fours’ after inspection, showing that an evaluation of all types of decisions (correct acceptance of training stimulus, correct rejection of unrewarded stimulus and the corresponding two types of errors) is tantamount [77–79]. In addition, the sequential nature of the inspection of the elements in a pattern yields certain predictions that can be tested further. For example, how does a bee trained to ‘two’ avoid accepting a ‘four’—even though the ‘four’ contains the required two elements? Must the bee then ascertain that a given pattern contains ‘more than two’ to reject it with certainty (for example, it appears in electronic supplementary material, video S1 that the bee rejects a ‘four’ after having inspected three items in the pattern)? What kind of flight manoeuvers and working memory strategies ensure that bees avoid counting an element twice?

## 7. Conclusion

Although traditionally regarded as a higher cognitive function, the ability to enumerate small sets of items is widespread in ‘lower vertebrates’ as well as mammals. Not even the basic architectural plan of the vertebrate brain seems to be required. Bees and most probably some other insects show a basic numerical competence, which may be limited to around four items. This is similar to the limit of the small number system of human adults and infants [31], non-human primates [78] and many other vertebrate species ([80]; see also Agrillo & Bisazza [4]). No evidence has yet been found for the existence of a separate number system for approximate processing of large numerosities in insects.

Basic numerical cognition, then, seems not to require (e.g.) a dedicated cortical module, but may instead be an inherent aspect of the process of organizing sensory input into objects of perception and maintaining object representations in working memory as required [31,40]. In support of this, theoretical and simulation studies show that relatively simple network models can mimic many experimental results on numerosity discrimination [6,53]. Basic numerical cognition does not seem to require a large brain [49]. Indeed, while small compared to vertebrate brains, insect brains appear to offer more than enough complexity. How the complex processing in the insect optic lobe is integrated with structures of the central brain to permit number-based visual discrimination remains largely unknown, but the diverse array of neuronal types and central projections (e.g. [61]) would seem to conceal more than enough complexity to implement simple classifier [60,81] and enumeration algorithms.

Although the small number system is often associated with parallel processing and perceiving the number of items in a small set ‘at a glance’ (subitizing), the brief stimulus exposures necessary to confirm this have only been used in primate experiments. Although parallel processing in working memory may be required, this does not necessarily mean that parallel processing at the visual input stage is also required. It may be informative to control presentation durations in experiments with lower vertebrates where stimuli are presented in parallel in numerosity discrimination tasks. Certainly, bees are unable to process visual scenes (other than the most basic visual attributes) when stimulus presentation duration is restricted [47]. Numerosity and other visual discriminations in bees instead seem to depend on serial processing [64], involving active scanning supported by a fast visual system [69]. The upper limit of small number perception more probably reflects the capacity limit of working memory, which may be similar across species in terms of number of representation that can be maintained, although large-brained species such as humans can represent more features of a given object [82]. Detailed analysis of the visuomotor behaviour underlying bees' choices in discrimination experiments is likely to elucidate the strategies (and also limitations) by which they make the perceptual choices they do.

## Data accessibility

This article has no additional data.
